# Effects of a Foot Placement Constraint on Use of Motor Equivalence during Human Hopping

**DOI:** 10.1371/journal.pone.0069429

**Published:** 2013-07-30

**Authors:** Arick G. Auyang, Young-Hui Chang

**Affiliations:** 1 School of Applied Physiology, Georgia Institute of Technology, Atlanta, Georgia, United States of America; University of California Merced, United States of America

## Abstract

Humans can robustly locomote over complex terrains even while simultaneously attending to other tasks such as accurate foot placement on the ground. We investigated whether subjects would exploit motor redundancy across the joints of the leg to stabilize overall limb kinematics when presented with a hopping task that constrained foot placement position. Subjects hopped in place on one leg (2.2 Hz) while having to place their foot into one of three target sizes upon landing (0.250, 0.063, 0.010 m^2^). As takeoff and landing angles are critical to this task performance, we hypothesized smaller target sizes would increase the need to stabilize (i.e., make more consistent) the leg orientation through motor equivalent combinations of segment angles. As it was not critical to the targeting task, we hypothesized no changes for leg length stabilization across target size. With smaller target sizes, we saw total segment angle variance increase due to greater signal-dependent noise associated with an increased activation of leg extensor muscles (medial and lateral gastrocnemius, vastus medialis, vastus lateralis and rectus femoris). At smaller target sizes, more segment angle variance was aligned to kinematic deviations with the goal of maintaining leg orientation trajectory. We also observed a decrease in the variance structure for stabilizing leg length at the smallest target conditions. This trade-off effect is explained by the nearly orthogonal relationship between the two goal-equivalent manifolds for leg length vs. leg orientation stabilization. Our results suggest humans increasingly rely on kinematic redundancy in their legs to achieve robust, consistent locomotion when faced with novel conditions that constrain performance requirements. These principles may generalize to other human locomotor gaits and provide important insights into the control of the legs during human walking and running.

## Introduction

As we move about our daily lives, our movements are subject to constant perturbations and constraints. Yet, the human locomotor system is robust and adaptable, allowing for quick and effective compensation to these perturbations to achieve stable locomotion. Kinematic motor redundancy allows access to a number of different joint configuration solutions for a given task goal even when the system is constrained. We have previously shown that certain task constraints such as hopping at non-preferred frequencies results in increased use of kinematic motor redundancy to stabilize leg length [10]. Our objective was to explore the robustness of this response by applying a constraint on foot placement that should increase need to stabilize leg orientation from cycle to cycle.

Human hopping provides an excellent model for the study of locomotion because the center of mass dynamics of bouncing gaits can be approximated by a low degree of freedom spring-mass model [1]–[4]. The low number of components that make up this model makes it useful for identifying potential limb level performance variables. Two kinematic variables in particular, leg orientation and leg length, are of particular interest as there exists both biomechanical and neurophysiological evidence for their control [3]–[9]. More recently, both leg orientation and leg length have been shown to be stabilized through the coordination of segment angles during human hopping [10]. As such, leg length and leg orientation provide task-level variables for testing the effects of task constraint during a hopping task. In the present study, we will maintain a definition of “stabilization” as the minimization of task-level variance (i.e., leg length and leg orientation) through the non-random partitioning of segment angle variance into goal equivalent and non-goal equivalent variance.

Task-level limb kinematics are robust to perturbations during legged locomotion. When confronted with environmental perturbations, such as differences in surface stiffness, subjects preserve a stable center of mass trajectory and effective leg stiffness [11]–[13]. Mechanically constraining individual joint kinematics and kinetics also yields invariant center of mass trajectories and effective leg stiffness [14], [15]. After neuromuscular injury, animals maintain invariant leg orientation or leg length trajectories in the injured limb despite significantly higher individual joint variability after injury [16]. These examples of perturbations are well controlled in a laboratory setting. However, natural locomotion is often affected by more than a single perturbation. How do the stabilizations of leg length and leg orientation change when constraints on foot placement are placed on both variables when both variables are critical for task completion?

By analyzing the kinematic variance over multiple cycles, we can examine if there is a non-random pattern to the structure of variance. Kinematic variables such as segment angles are subject to intrinsic cycle-to-cycle variability, however, metrics such as leg length and leg orientation reveal comparatively low variability [10]. This suggests the intrinsic cycle-to-cycle variability of segment angles can be partitioned to minimize variability of leg length and leg orientation [10]. Investigating hopping kinematics through the framework of the Uncontrolled Manifold (UCM) analysis has previously shown the partitioning of segment angle variance to stabilize leg length and leg orientation at specific times in the hopping cycle [10]. We observed that kinematic variance is partitioned into variability that causes change in the leg length or leg orientation and variability that does not cause change. The partitioning of this data creates a variance structure that can describe whether the task variable is being stabilized. In addition to the intrinsic variability observed under preferred conditions, increasing hopping frequency increases the total segment angle variance [10]. By observing how the locomotor system partitions this increase in kinematic variance, we can gain some insight into how the locomotor system compensates to an imposed task constraint.

The purpose of this study was to investigate how humans coordinate and adjust limb segment angles to stabilize overall leg length and leg orientation when presented with locomotor tasks of varying constraints. Specifically, we limited the landing area of our hopping subjects by projecting one of three target sizes on the ground. We predicted that stabilizing leg orientation would be more important than stabilizing leg length for hopping into the smaller targets. Since cycle to cycle deviations in leg orientation would lead to foot placements outside the target area, we hypothesized that segment angle variance would be more partitioned to not affect leg orientation when presented with smaller target sizes. As cycle-to-cycle consistency of leg length would not help place the foot into smaller targets, we hypothesized that changes in target size would have no effect on the variance structure of limb segment variance acting to stabilize leg length.

## Methods

### Subjects

All subjects gave their written informed consent prior to participating in this study according to a protocol approved by the Georgia Institute of Technology's Human Subjects Institutional Review Board and in accordance with the principles expressed in the Declaration of Helsinki. Eleven healthy human subjects with no prior history of lower extremity injuries volunteered for this study (6 males, 5 females, mean±SD age  = 27±5 years, mass  = 60.5±10.1 kg, left leg length  = 86.48±5.5 cm, right leg length  = 86.33±5.49 cm, height  = 169.38±9.27 cm).

### Experimental Protocol

We made anatomical measurements and placed eight retroreflective markers on anatomical landmarks on each of the lower extremities using a modified Helen Hayes marker set (anterior superior iliac spine, posterior superior iliac spine, thigh segment, lateral femoral epicondyle, shank segment, lateral malleolus, head of the second metatarsal phalangeal joint, and calcaneus). Based on these marker positions, ankle, knee and hip joint centers were calculated using Vicon Plug-in Gait Model (Vicon Motion Systems; Los Angeles, CA). Subjects hopped in place on their right leg at 2.2 Hz for three trials per target condition (large, medium, and small). Hopping frequency was determined based on previously reported preferred hopping frequencies [10], [17]. The presentation order of the target conditions was randomized for each subject. Subjects matched the prescribed hopping frequency to the beat of an audible metronome. A minimum thirty-second practice trial was provided to become familiar with hopping at the prescribed frequency and target conditions prior to collection of test trials. We visually confirmed that subjects were able to make contact with the beat of the metronome. Each data collection trial lasted approximately thirty seconds. Each subject stood in the center of the target on a force platform and crossed their arms over their chest and hopped on their right leg. Subjects were instructed to hop with a strategy consistent with if they had to perform the task for a long period of time while trying to land in the target. Approximately 190 hops were analyzed per subject per target condition.

### Target Sizes

Targets were projected onto the platform from above. We used three square targets with sizes 0.250 m^2^ (0.5 m×0.5 m), 0.063 m^2^ (0.25 m×0.25 m), and 0.010 m^2^ (0.1 m×0.1 m).

### Kinematics and Kinetics

We used a five-camera motion-analysis system (120 Hz; Vicon Motion Systems; Los Angeles, CA) to capture kinematic marker data. We filtered data using a zero phase shift fourth-order Butterworth low-pass filter with a 10 Hz cut-off frequency. We calculated ankle, knee, and hip joint centers to create a linked segment kinematic model. We then calculated four sagittal plane segment angles: toe to ankle (foot segment), ankle to knee (shank segment), knee to hip (thigh segment), and hip to anterior superior iliac spin (pelvis segment) with respect to the ground. We determined ground contact and liftoff events using a force platform (1080 Hz; AMTI; Watertown, MA) by detecting when the vertical ground reaction force crossed a threshold of 32 N. Total segment angle variance is calculated as the sum of the individual segment angle variances. Total segment angle variance for each condition was normalized to the total segment angle variance from the large target condition for each subject.

We performed inverse dynamics to calculate torques about the ankle, knee, and hip joints using sagittal plane kinematics and force data. The inertial properties of the segments were estimated based on subject anthropomorphic measurements [18]. Joint torque impulse was calculated as the integral of the joint torque curve over stance phase. Joint torque impulses were normalized for each subject based on subject weight and the anatomical leg length measured from the anterior superior iliac spine to the medial malleolus.

### Uncontrolled Manifold Analysis

We defined leg length as the magnitude of the leg vector and leg orientation as the angle of the leg vector relative to horizontal where the leg vector is defined from the toe marker to the ASIS marker. Two geometric mathematical functions related the foot, shank, thigh, and pelvis segment angles to either leg length or orientation. These functions were linearized to create two Jacobian models. The first model related changes in the magnitude of this leg vector to changes in the segment angles while the second model related changes in the orientation of the leg vector to changes in segment angles [10].

The Uncontrolled Manifold (UCM) hypothesis has been described in previous studies [10], [19], [20]. We will be using definitions and methods similar to those described in Auyang et al 2009, which we briefly explain here [10]. The UCM is a linearization of each of our mathematical functions and is estimated as the null space (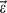) of the Jacobian (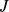) of each function relative to a static reference leg posture (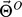, Eq. 1).
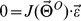
(1)


The UCM analysis is a static analysis that is performed over successive hop cycles at a specific instant in time of the cycle and with respect to a reference leg posture. We performed the UCM analysis over successive hops at 1% increments of the hopping cycle. In each of our 100 UCM analyses, we defined our reference leg posture (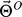) as the average set of segment angles at that specific time in the hopping cycle. We then projected the deviations of the segment angles (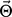) from this reference posture onto the null space (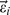) to resolve the fraction of deviations that did not affect the task, i.e. goal equivalent deviations (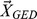, GED). The remaining fraction was then deemed to be orthogonal to the null space and, hence, non-goal equivalent deviations (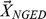, NGED, Eq. 3). Each component was normalized by the degrees of freedom parallel (*n*) and orthogonal (*d*) to the UCM.

Respectively, these are referred to as goal equivalent variance (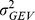; Eq. 2):
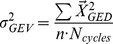
(2)


and non-goal equivalent variance (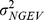; Eq. 3).
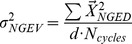
(3)


Due to differences in total variability between subjects, we normalized our variance measures by calculating the Index of Motor Abundance (*IMA*), a metric of the amount of motor abundance that is selectively utilized to stabilize the performance variable [10], [21]. An IMA greater than zero indicates that more segment angle variance is partitioned from hop to hop to minimize any destabilizing effect on leg length and orientation. An IMA equal to zero indicates that there was equal partitioning of segment angle variance to stabilize leg length and orientation. An IMA less than zero indicates that the majority of the small deviations occurring at the level of the limb segments acted to destabilize leg length or orientation. We calculated the IMA as (Eq. 4):
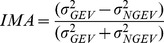
(4)


We calculated 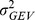, 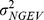 and IMA at 1% intervals during the contact and aerial phases of the hopping cycle for leg length and orientation control functions at each of the three target sizes: large, medium, and small. Data were first divided into stance and aerial phase for each subject and then further divided into ten equal bins of 10% of each respective phase. Note that the sizes of the bins in the aerial phase are smaller than those in the stance phase. All above calculations were performed using custom software coded in Matlab (Mathworks, Natick, MA).

### Electromyography

We did a post-hoc collection of electromyography (EMG) data from five of our original subjects. The protocol was repeated as described above except with the collection of EMG from seven muscles of the right leg: tibialis anterior (TA), lateral gastrocnemius (LG), medial gastrocnemius (MG), vastus lateralis (VL), vastus medialis (VM), rectus femoris (RF), and the long head of the biceps femoris (BF). Data were collected using a wireless EMG system (1080 Hz, Noraxon TeleMyo 2400T G2). EMG data were processed using custom software coded in Matlab (Mathworks, Natick, MA). EMG data were band pass filtered from 10–500 Hz, rectified, and low pass filtered at 10 Hz. EMG data for each channel were normalized to the peak activity recorded during the large hopping condition for that channel, respectively. Onset and offset of EMG activity for each muscle was determined as activity two standard deviations above the mean activity observed during the quiescent period (i.e. typically in the aerial phase). Burst duration and amplitude were calculated for each muscle.

### Statistical Analysis

A Student's one sample t-test (α = 0.05) was used to test whether normalized total variance of segment angles changed with target size. The same test (α = 0.05) was used to test for significant differences in GEV and NGEV during hopping in the small and medium target conditions with respect to the large target condition. To determine whether a performance variable was stabilized by local variables at each 10% of the hopping cycle, we performed a Student's one-sample, one-tailed t-test (α = 0.05) to test whether IMA was significantly greater than 0. To test whether changes in target size are associated with changes in IMA, we used a repeated measures analysis of variance (ANOVA) to test for changes in average IMA across target size (α = 0.05) and a post-hoc test with Bonferroni correction to determine which targets had different IMAs. Our within subject variable was target size with three levels: small, medium, and large. A bivariate linear correlation was used to test whether there was a linear relationship between leg orientation and leg length IMA. We used a repeated measures analysis of variance (ANOVA) to test for the effect of target size, our within subject measures, on mean EMG activity and EMG burst duration of each individual muscle. All statistical analyses were done using SPSS software (SPSS Inc.; Chicago, IL).

## Results

### Kinetics

Normalized joint torque impulses showed no statistical difference between different target conditions (n = 11). Ankle extensor torque impulses for large (0.176±0.026), medium (0.178±0.031), and small (0.177±0.027) targets showed no statistical difference (p = 0.988). Knee extensor torque impulses for large (0.0534±0.012), medium (0.049±0.02), and small (0.054±0.019) targets showed no statistical difference (p = 0.786). Hip extensor torque impulses for large (0.033±0.011), medium (0.018±0.015), and small (0.027±0.021) targets showed no statistical difference (p = 0.086).

### UCM Results

**Figure 1:**
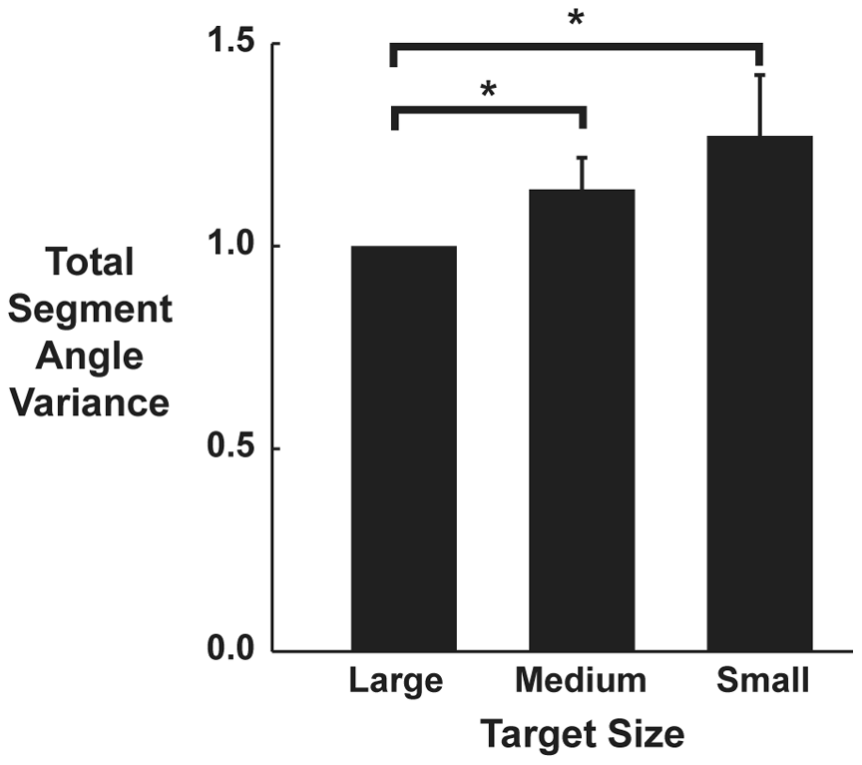
Total variance increases with smaller target sizes. Total segment angle variance normalized to large target condition. Bars are the averaged total variances for all subjects with ±1 standard deviation. *denotes significant difference (p<0.05).

**Figure 2:**
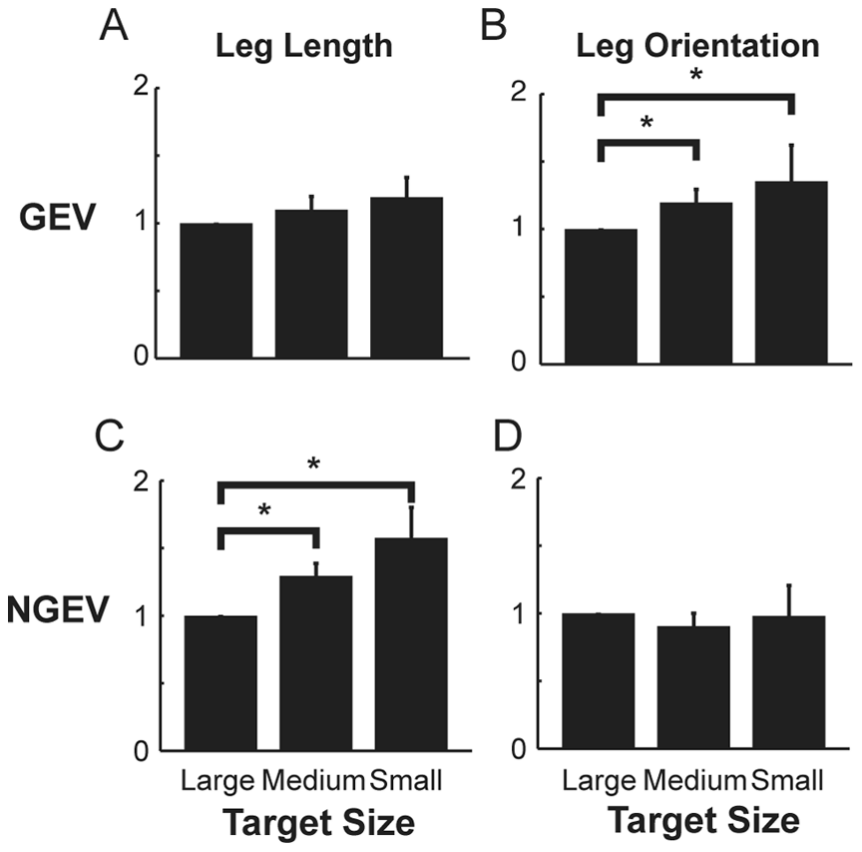
Variance components as a function of target size. Goal equivalent variance (GEV) and non-goal equivalent variance (NGEV) normalized to large target condition for the three target conditions for (a & c) leg length and (b & d) leg orientation. Bars are the averaged total variances for all subjects with ±1 standard deviation. Leg length NGEV and leg orientation GEV increased for medium and small target condition relative to large target condition. *denotes significant difference (p<0.05).

We normalized total segment angle variance relative to the large target condition for the medium and small target conditions (n = 11). Total segment angle variance significantly increased with smaller targets (p≤0.05, Figure 1). GEV and NGEV components of leg length and leg orientation were also normalized to the large target condition for the medium and small target conditions (n = 11, Figure 2). For leg orientation, the NGEV did not change with smaller targets, while the GEV significantly increased with smaller target sizes (p≤0.05, Figure 2b and 2d). For leg length, we observed the opposite trend, with no change in GEV and a significant increase in NGEV with smaller targets (p≤0.05, Figure 2a and 2c).

**Figure 3:**
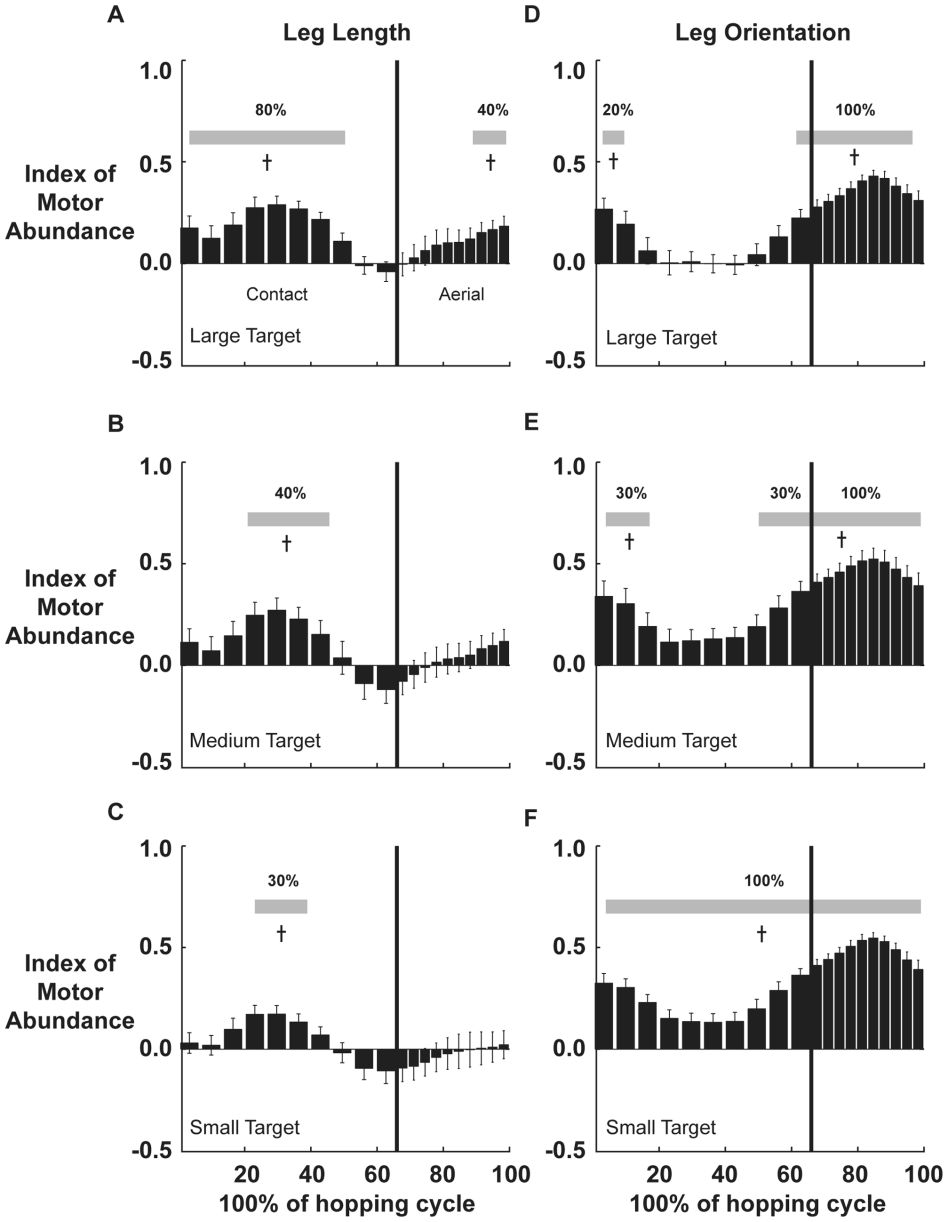
Variance structure over the hop cycle. 3. Index of Motor Abundance (IMA) (a–c) for leg length stabilization across three target conditions. (d–f) IMA for leg orientation stabilization across three target conditions. Bars are the averaged IMA for 10% intervals in contact and aerial phase. (n = 11, ±1 standard deviation). The duration of leg length stabilization decreased with decreasing target size but remained stabilized at midstance. The duration of leg orientation stabilization increased with decreasing target size. Gray bars denote period where IMA was significantly greater than 0 (p<0.01).

**Figure 4:**
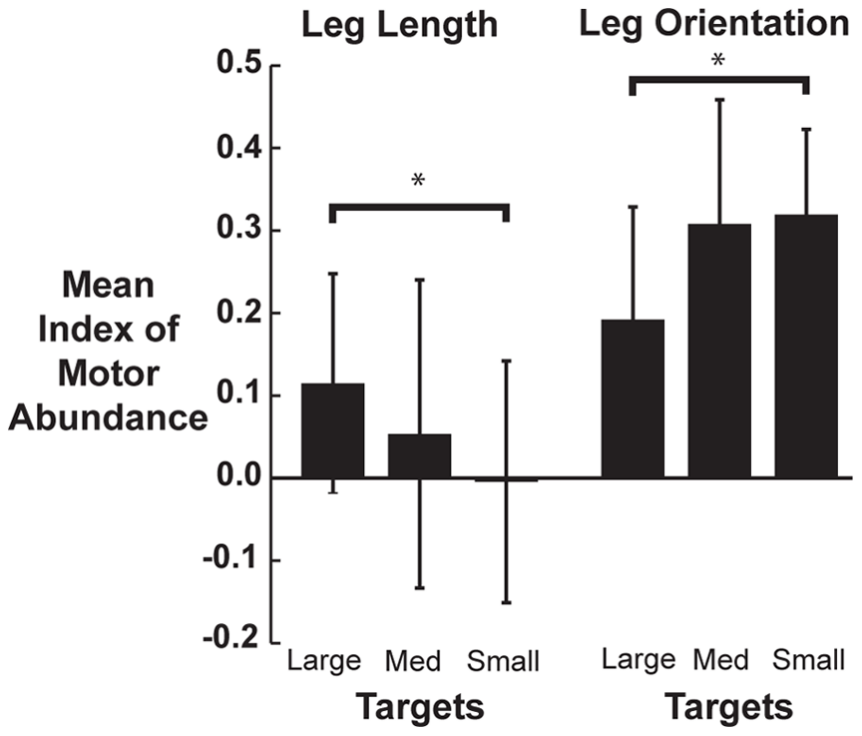
Variance structure as a function of target size. Hop cycle averaged Index of Motor Abundance (IMA) for leg orientation and leg length for the three target conditions. Bars are the averaged IMA across all subjects and bins with ±1 standard deviation. Average stabilization of leg length decreased from large to small target while leg orientation stabilization increased from large to small target. *denotes significant difference (p<0.05).

In the large target condition, leg length showed significant stabilization during most of stance (0–80% of stance) and late aerial phase (70–100% of aerial phase, p<0.01). Peak stabilization occurred at midstance (p≤0.01, Figure 3a). As target size decreased, the period of leg length stabilization decreased to 30–70% of stance in the medium target condition (Figure 3b) and 40–60% stance for the small target (p≤0.01, Figure 3c). Peak stabilization remained at midstance. Mean leg length IMA averaged over the entire hop cycle decreased with smaller targets and significantly decreased between the large and small target conditions (F(2,30)  = 4.938, p = 0.03, Fig. 4).

In the large target condition, leg orientation was significantly stabilized during 0–20% and 90–100% of stance and all of aerial phase (p≤0.01, Figure 3d). Peak stabilization occurred at mid-aerial phase. As target size decreased to the medium target, the period of leg orientation stabilization increased to 0–30% and 70–100% of stance in addition to all of aerial phase (p≤0.01, Figure 3e). With the small target, we saw significant stabilization of leg orientation throughout the entire hopping cycle (p≤0.01, Figure 3f). Average leg orientation IMA showed an increasing trend with smaller targets and showed a significant increase between the large and small target conditions (F(2,30)  = 4.675, p = 0.02, Figure 4). A sensitivity analysis showed that leg orientation was most sensitive to changes in shank and thigh angles. The average coefficients ± SD for the leg orientation Jacobian for the foot, shank, thigh, and pelvis segments were: 0.08±0.012, 0.36±0.05, 0.46±0.04, and 0.07(0.016) respectively.

**Figure 5:**
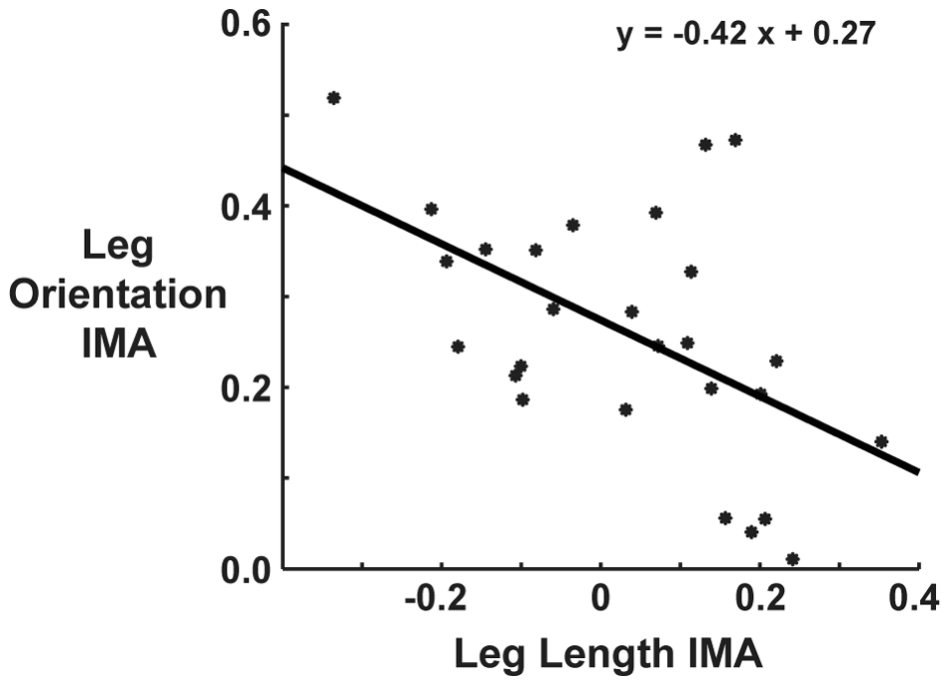
Relationship of leg orientation versus leg length stabilization. IMA's for leg orientation stabilization and leg length stabilization for all subjects and conditions showed a significant negative linear correlation (p = 0.03). Increases in leg orientation IMA with decreases in leg length IMA corresponds with smaller target sizes.

Leg length and leg orientation IMAs showed a significant negative linear correlation (r^2^  = 0.527, p = 0.03, Figure 5). The dot product of the leg length and leg orientation null spaces showed they were at an orientation of 78°–85° relative to each other throughout the hopping cycle. This indicates that the null spaces of the Jacobians for leg length and leg orientation stabilization (in segment angle space) were nearly orthogonal.

### Task performance

The standard deviation of the anterior posterior foot placement for large, medium and small targets were 0.185±0.0382 m, 0.187±0.0364 m, and 0.182±0.0347 m respectively. The standard deviation of the anterior-posterior foot placement error during the contact phase showed no statistical difference (p = 0.08). Hop height for the small, medium, and large targets were 2.6 cm±1.2 cm, 2.7 cm±1.1 cm and 2.5 cm±1.0 cm. There was no statistical difference (p = 0.35).

### EMG

**Figure 6:**
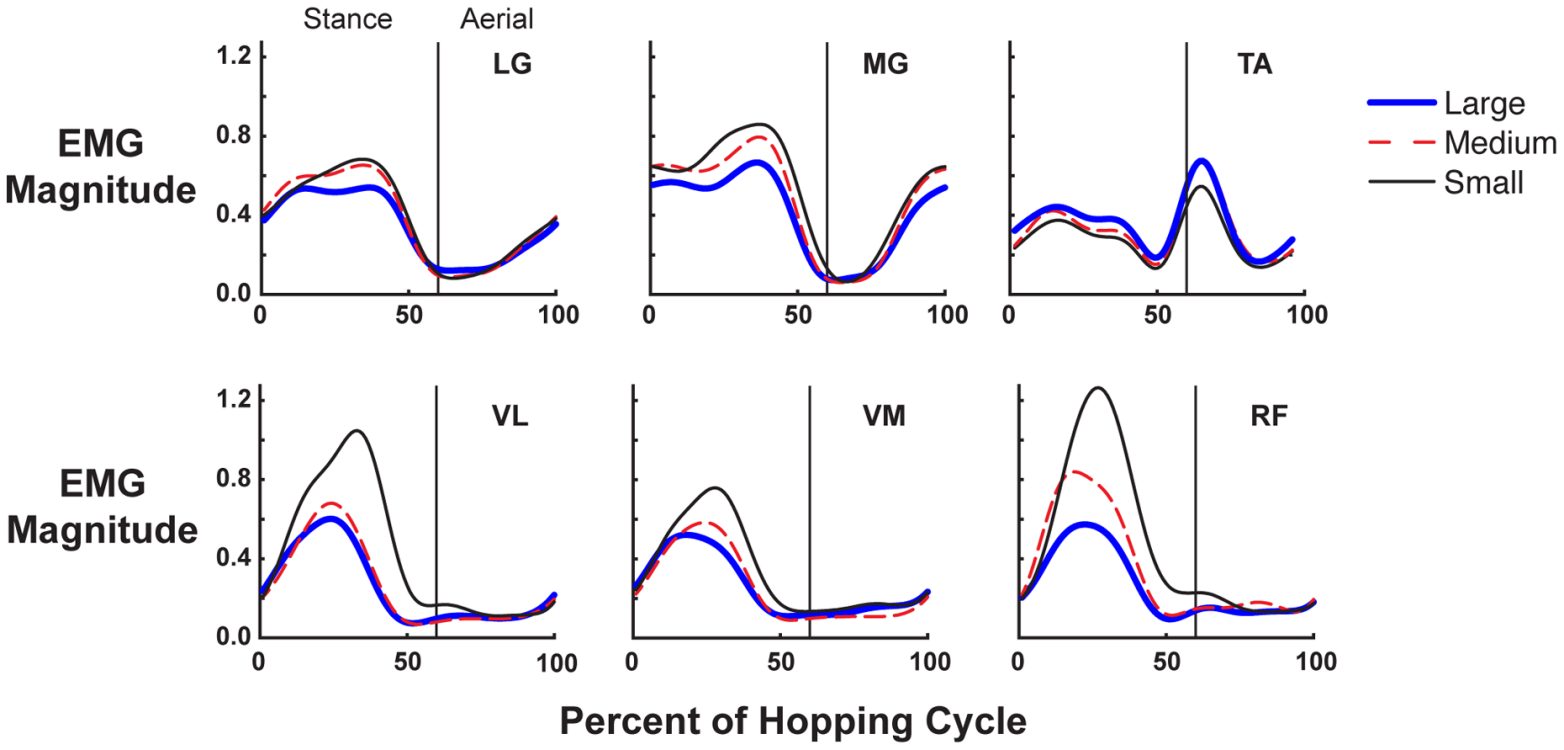
Muscle activity over time. Average normalized EMG activity for LG, MG, VL, VM and RF across the three target conditions (large: thick blue lines; medium: red dashed lines; small: thin black lines) for 100% of the hopping cycle (n = 11). Muscle activity generally increased with smaller target conditions.

**Figure 7:**
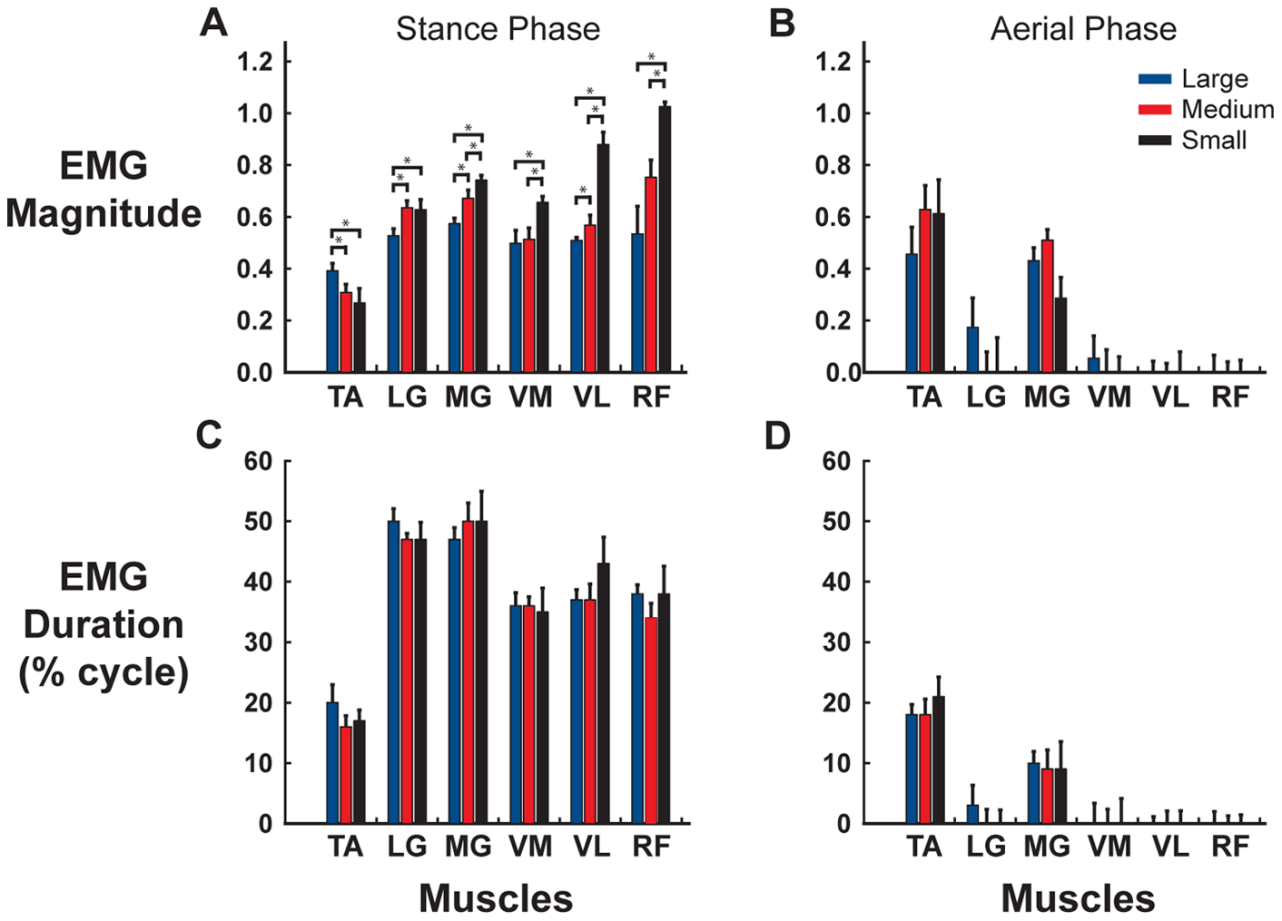
Mean muscle activity as a function of target size. EMG burst activity magnitude (a–b) and EMG burst duration (c–d) during stance and aerial phase for the three targeting conditions (large: blue, left; medium: red, middle; small: black, right). Magnitude of activity increased for all muscles in smaller, more smaller target sizes compared to the large, easier condition except for TA, which showed a significant decrease. No changes were observed for burst duration. *denotes significant difference (p<0.05).

Peak activation of all muscles recorded, except tibialis anterior, occurred at approximately mid-stance (Figure 6). During stance phase, there was a significant increase in mean muscle activity of the LG, MG, VM, VL, and RF muscles with smaller targets (F(2,12) = 5.43, 6.21 5.95, 6.34, and 6.12 respectively; p≤0.05). Mean TA activity decreased during stance phase with smaller targets (F(2,12) = 4.98; p≤0.05, Figure 7). There was no statistical change in mean BF activity across all target conditions (p>0.05). There were no significant changes in mean muscle activity during aerial phase in any of the muscles across target conditions (Figure 7b). The onset, offset and duration of muscle activity during both stance (Table 1 and Figure 7c) and aerial phase (Figure 7d) for all muscles recorded showed no significant changes with target condition.

**Table 1:** Mean of EMG activity onset, offset, and duration during stance.

Muscle	Target Size	Onset (%cycle)	Offset (%cycle)	Duration (%cycle)
**TA**	*Large*	7	27	20
	*Medium*	7	23	16
	*Small*	9	26	17
**G**	*Large*	1	51	50
	*Medium*	2	49	47
	*Small*	1	48	47
**MG**	*Large*	1	48	47
	*Medium*	1	51	50
	*Small*	1	51	50
**VM**	*Large*	1	37	36
	*Medium*	6	42	36
	*Small*	8	43	35
**VL**	*Large*	3	40	37
	*Medium*	5	42	37
	*Small*	4	47	43
**RF**	*Large*	2	40	38
	*Medium*	6	40	34
	*Small*	6	44	38
**BF**	*Large*	7	47	40
	*Medium*	11	47	36
	*Small*	9	51	42

## Discussion

In this study we explored how the neuromechanical system coordinates and controls pelvic limb segments to stabilize leg orientation and leg length during human hopping when the task is more constrained. We used three different target sizes to constrainfoot placement. We used a UCM analysis to determine whether leg length or orientation was stabilized through partitioning of segment angle variance. The control condition IMA data of our current study, hopping at 2.2 Hz in the large target was similar to hopping in place with no target when compared with previous IMA findings for 2.2 Hz [10]. Peak periods of stabilization for leg orientation and leg length occurred at similar points in the hopping cycle as with no target hopping (Figure 3a and 3d, Auyang et al. 2009). By constraining foot placement through smaller target sizes, we placed greater importance on achieving an invariant leg orientation trajectory and less importance on having a consistent leg length.

### Effects of task constraints on performance variable stabilization

Leg orientation and leg length are low degree of freedom performance variables that are stabilized through a high dimensional kinematically redundant set of limb segments. In turn, deviations of the segment angles can have a significant effect on leg orientation and leg length trajectories. To approach having invariant limb kinematics over many hop cycles, one strategy could be to minimize the total variance of all segment angles when the task is made more constrained [22], [23]. Our subjects, however, actually increased total segment angle variance when target size decreased (Figure 1). Another strategy to achieve consistency in a performance variable is to partition the variance of elemental variables such that goal-equivalent variance is favored [19], [22], [24]. We found significant changes with the segment angle variance structure as target size decreased. Supporting our hypothesis, more segment angle variance was partitioned as target size decreased to allow deviations that would not affect leg orientation (Figure 4b). We also hypothesized no change in leg length IMA with decreasing target size because leg length should not have been affected by the increased constraint in foot placement precision. We rejected this hypothesis, however, since the average IMA for leg length stabilization actually decreased with smaller target sizes (Figure 4a). This decrease is likely due to the orthogonality of the leg orientation UCM space relative to that of the leg length.

The changes and differences in leg length and leg orientation IMA can be attributed to the relative amounts of segment angle variance partitioned into Goal-Equivalent Variance (GEV) and Non-Goal-Equivalent Variance (NGEV). The increase in leg orientation IMA with smaller targets was due solely to an increase in the GEV component (Figure 2b) and no change in the NGEV (Figure 2d) component. This means that the increases in total segment angle variance with smaller target sizes (Figure 1) acted to stabilize leg orientation. In contrast, for leg length, the increased segment angle variance was a result of an increase in the NGEV component (Figure 2c) while not affecting the GEV component (Figure 2c), directly resulting in greater deviations in leg length from hop to hop.

Hopping in a target area that is much larger than the average hopping distribution observed during non-targeted hopping requires very similar control [10]. Peak stabilization for leg orientation and leg length occurred at mid-stance and mid-aerial phase respectively during the large target hopping condition. The magnitude and timing of peak stabilization for both did not change as target size decreased (Figure 3). Leg length was stabilized throughout most of the hopping cycle while leg orientation was stabilized at the beginning and end of stance phase and all of aerial phase. These findings are qualitatively similar to the leg orientation and leg length IMA profiles found in hopping in place at 2.2 Hz with no target [10].

Despite the overall decrease of leg length stabilization as target size decreased, leg length stabilization persisted at mid-stance. As target size decreased, leg length IMA generally decreased across the entire hopping cycle (Figure 3a–c). This resulted in increasingly shorter periods of time in which leg length was stabilized. Yet, despite the overall decrease in IMA, peak stabilization of leg length persisted at mid-stance (40–60% of stance) even at the most constrained condition (small target). Mid-stance is an important period to stabilize leg length during hopping in place because it is then that leg length is most sensitive to small deviations of segment angles [10]. It is also the point in the hopping cycle where there is maximum joint flexion and when peak forces are generated on the ground [17]. A more flexed posture decreases the effective mechanical advantage of the muscles crossing the joints and would require higher muscle forces to generate the same ground reaction force [25], [26]. The stabilization of leg length at midstance likely limits costly deviations in peak joint moments when forces are at their peak. The results of this study provide further support for the importance of leg length stabilization at midstance previously observed during human hopping [10].

For leg orientation, as target size decreased, IMA generally increased across the entire cycle except around the mid-aerial phase where it remained at a peak (Figure 3d–f). Control of leg orientation during aerial phase is important for foot placement at ground contact, which determines forward velocity and spring-loaded inverted pendulum dynamics [27]. As target size decreased and more precise foot placement became necessary to land in the target, anticipatory adjustments in the aerial phase alone may not have been enough to precisely land in the smaller targets. It may have been necessary to minimize deviations of leg orientation throughout the entire hop cycle in anticipation of the next foot placement. Leg orientation during the stance phase largely determines the ballistic dynamics of the center of mass during aerial phase [3], [4], [28]. The ballistic dynamics are an important contributor to the center of mass position at landing and likely explain the increased IMA for leg orientation during the stance phase.

Surprisingly, despite changes in GEV, NGEV, and IMA metrics, we saw no differences in the anterior/posterior foot placement during landing. In other words, while there were changes in how the joint angle variances were partitioned across hopping conditions, there was no actual change in the performance of foot placement. This presented a rather interesting result in that the presentation of smaller targets resulted in greater joint angle variance, greater variance structure for stabilizing leg orientation and increased muscle activity, without any improvement in the task goal. The exact reasons for this result are not yet clear and would require further investigation to uncover the causes.

### Muscle activations for leg orientation stabilization

For the task of hopping in place, regardless of target size, leg orientation is over three times more sensitive to changes in shank and thigh angles, the two longest segments, than to changes in the foot and pelvis angles. This means that for a given amount of angular change in all the joints, errors in shank and thigh angles will translate to the largest deviation of leg orientation. To successfully stabilize leg orientation as target size decreased, control of the shank and thigh segment angles, or the knee joint angle, is critical.

Given the increased task demands and the sensitivity of leg orientation to the knee joint angle, to stabilize leg orientation, one strategy might be to stiffen the knee joint through increased co-contraction of muscles across the knee. Since we observed increased stabilization of leg orientation throughout stance, we might expect an increase in antagonistic muscle activity across the knee for increased joint stiffness. Co-contraction across individual joints to increase stability of a limb level performance variable during novel tasks has been observed in a variety of studies [29]–[33]. We found a significant increase in EMG amplitude with smaller target size of all recorded extensor muscles. The onset of bursting activity is consistent with EMG recordings from similar muscles in previous hopping studies [33]–[37]. Despite the increased activity of LG, MG, VL, VM, and RF muscles, net extensor joint torques for all joints did not increase with smaller target sizes. There was also co-activation of the antagonistic knee flexor muscles MG and LG with knee extensor muscles VL and RF as the period of activation overlapped across all task conditions. In other locomotion tasks where increased knee stabilization is required, a similar co-contraction strategy was observed between the quadriceps and gastrocnemius muscles [38].

An alternative explanation for increases in EMG activity of the vasti, rectus femoris, and gastrocnemius muscles may be for mediolateral stabilization. These muscles are typically considered to function in the sagittal plane, however, they have been shown to make significant contributions to frontal plane movements [39], [40]. Though it is outside the scope of the present study, future work could distinguish between these explanations through a set of constraints that do not require mediolateral stabilization. A lack of change in EMG in this case would support the hypothesis that the increased muscle activity seen in the current study is for mediolateral stabilization.

The exact reason for the observed increases in EMG amplitude is not clear and warrants further investigation. The increased muscle activity we saw is attributable to either increased neural drive or increased gains in reflex pathways. We speculate that the observed increase in muscle activity is due to increased cortical activation. Motor cortex neuronal activity in locomoting cats show significant increases in activity when accurate foot placement was required [41], [42]. In human studies, accurate foot placement during a seated knee extension task resulted in increased cortical activity compared to when accuracy was not required, despite no change in task performance [43]. In our study, we saw an increase in IMA for leg orientation, increased EMG amplitude, but no change in the anterior posterior foot placement variability between target conditions. Regardless of the cause, the increase in muscle activity resulted in an increase in signal-dependent motor noise in segment kinematics [44]. The additional segment angle variance due to this increased motor noise was partitioned along the leg orientation manifold within the kinematic task space.

### Implications on completion of accurate hopping

There is a significant negative correlation between leg orientation IMA and leg length IMA as target size decreased, which suggests a trade-off effect between stabilization of leg orientation and leg length (Figure 5). Consequently, the GEV component for leg length largely occupies the NGEV space of leg orientation and vice versa. Although the spaces are nearly orthogonal, this does not preclude simultaneous stabilization, as there exists an intersecting solution space, where the simultaneous stabilization of both leg orientation and leg length can be accomplished. For instance, this is exemplified at mid-stance when hopping in the small target condition (Figure 3c & f). One strategy for limb control is to simultaneously stabilize all performance variables throughout the entire task, e.g. this type of control has been implemented in some robotic systems [45], [46]. This is a conservative strategy that greatly restricts available limb kinematics. Alternatively, our results suggest that biological systems maximize the available solution space by only stabilizing performance variables at key times when they are critical to each task [10], [21], [47].

Like these artificial robotic systems, humans are capable of stabilizing both leg orientation and leg length simultaneously; however, humans do not do so unless the task requires it. This supports the idea that the neuromuscular system favors a “control as needed” strategy to maximize redundancy rather than a “control always” strategy. A similar result has been show in vertical and horizontal force control during hopping [21] and in finger force production [48]. Maximizing motor redundancy in this way may be a strategy for allowing the biological system to be more adaptable during locomotion. The ability to partition variance in response to specific task constraints allows for more precise compensation for new perturbations and makes us more robust to changing conditions in the environment. This could allow, for example, the negotiation of an obstacle while maintaining stable locomotion.

## Conclusions

A concomitant increase in partition of segment angle variance to stabilize leg orientation was observed with a decrease in segment angle variance structure to stabilize leg length stabilization as target size decreased. Despite being able to simultaneously stabilize both leg orientation and leg length as seen during midstance, both performance variables were not simultaneously stabilized throughout the hopping cycle. This suggests that humans try to maximize the kinematic solution space during locomotion by only stabilizing leg orientation and leg length at respective critical times during the gait cycle. The locomotor system maximizes the local variable operational space by stabilizing performance variables as needed, which provides the neuromuscular system with greater flexibility to compensate for unexpected changes as locomotor task constraints increases.
